# Influence of surface treatment on PEDOT coatings: surface and electrochemical corrosion aspects of newly developed Ti alloy†

**DOI:** 10.1039/c8ra01718b

**Published:** 2018-05-24

**Authors:** A. Madhan Kumar, M. A. Hussein, Akeem Yusuf Adesina, Suresh Ramakrishna, N. Al-Aqeeli

**Affiliations:** Centre of Research Excellence in Corrosion, Research Institute, King Fahd University of Petroleum and Minerals Dhahran Saudi Arabia madhankumar@kfupm.edu.sa +966-538604818 +966-538801789; Department of Mechanical Engineering, King Fahd University of Petroleum and Minerals Dhahran 31261 Saudi Arabia; Graduate School of Biomedical Science and Engineering, Hanyang University Seoul South Korea; College of Medicine, Hanyang University Seoul South Korea

## Abstract

Surface treatment of metallic materials prior to the application of polymer coatings plays an important role in providing improved surface features and enhanced corrosion protection. In the current investigation, we aimed to evaluate the effect of surface treatment of newly developed TiNbZr (TNZ) alloys on the surface characteristics, including the surface topography, morphology, hydrophobicity and adhesion strength of subsequent poly(3,4-ethylenedioxythiophene) (PEDOT) coatings. The surface morphology, chemical composition, and surface roughness of both treated and coated alloys were characterized by scanning electron microscopy, energy dispersive spectroscopy, and optical profilometry, respectively. The adhesion strength of the coating was measured using a micro scratch machine. Furthermore, we also evaluated the performance of electrochemically synthesized PEDOT coatings on surface-treated TNZ alloys in terms of the surface protective performance in simulated body fluid (SBF) and *in vitro* bioactivity in osteoblast MG63 cells. Surface analysis findings indicated that the nature of the PEDOT coating (surface morphology, topography, wettability and adhesion strength) was intensely altered, while the surface treatment performed before electrodeposition facilitated the overall performance of PEDOT coatings as implant coating materials. The obtained corrosion studies confirmed the enhanced corrosion protection performance of PEDOT coatings on treated TNZ substrates. *In vitro* cell culture studies validated the improved cell adhesion and proliferation rate, further highlighting the important role of surface treatment before electrodeposition.

## Abstract 2

Surface treatment of metallic materials prior to the application of polymer coatings plays an important role in providing improved surface features and enhanced corrosion protection.

## Graphical abstract

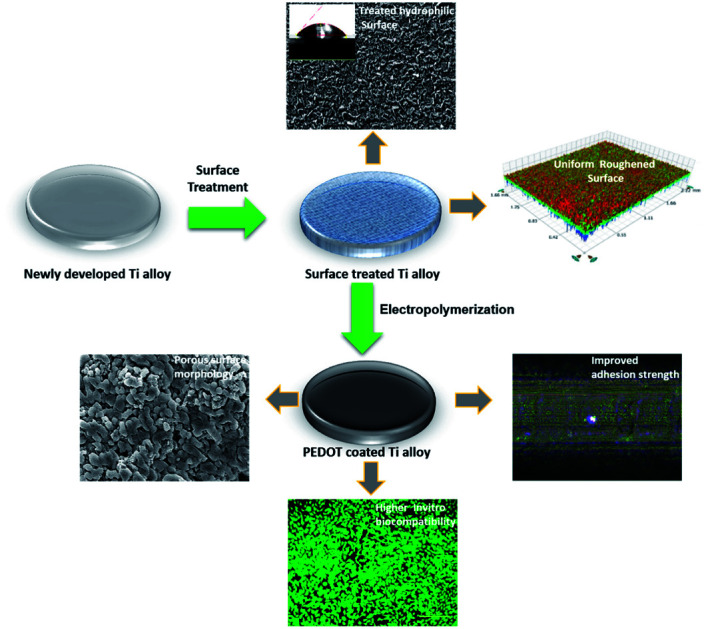

## 1 Introduction

Titanium (Ti) and its alloys have been the subject of widespread research owing to their desired properties, such as reasonable specific strength, significant ductility, good biocompatibility and better corrosion resistance compared to stainless steel and Co alloys.^1^ The desired characteristics have established Ti alloys as promising implant materials for orthopedic applications.^2,3^ However, one of the main concerns regarding Ti alloys in implant applications is that they fail to interact with the adjacent bone in the initial implantation period. This behavior can lead to bone resorption near the implant material, hence increasing the possible threat of loosening when the implant is used for a prolonged period.^4–6^ In the past few decades, numerous surface modifications and coatings have been dedicated to making Ti surfaces bond chemically with human bone to improve corrosion resistance in physiological environments.^7–9^

In recent decades, conducting polymers (CPs) have attracted significant attention in the field of biomedical applications, specifically being explored as coating materials for metallic implants due to their versatile properties.^10–12^ Among the available CPs utilized for clinical applications, poly(3,4-ethylenedioxythiophene) (PEDOT) has drawn wide interest in the biomedical field due to its unique environmental stability as well as noticeable biocompatibility, easy synthesis with less expensive route, high charge mobility and thermal stability with high electrical redox properties.^13,14^ Moreover, among the available CPs, only PEDOT is manufactured industrially and traded globally for numerous applications.^15^ It has been recently indicated that the aqueous compatibility and biocompatibility of PEDOT are good in physiological environments and that the polymerization of EDOT molecules has also been directly performed in physical muscles due to its high biocompatibility arising from structural resemblance with natural constituents similar to melanin.^16^ PEDOT coatings showed cytocompatibility and promoted human serum albumin adsorption and cell adhesion.^17^

As a main strategy to synthesize PEDOT, electrochemical polymerization has numerous distinctive benefits among the available routes, including lower quantities of required monomers, single stage preparation of conducting polymer layers, accessibility of characterization and ease of adjusting the thickness of the polymer coating. Gopi *et al.* have synthesized PEDOT composite coatings on 316L SS implants through electrochemical route and the prepared coatings displayed promising result in *in vitro* biological studies.^18^ Furthermore, the authors have also prepared the copolymer of PEDOT coatings on 316L SS implants using electrochemical approach and copolymer processed with 50 : 50 feed ratio exhibited high corrosion protection behavior with improved cell growth on MG63 osteoblast cell.^19^ Recently, K. Catt *et al.* reported the preparation of PEDOT composite coatings on Mg implants using electrochemical deposition and described the effective role of PEDOT coatings as anti-corrosion coatings on Mg implants using multiple mechanisms.^20^ However, the main obstacle in the electrodeposition of conducting polymers on metallic materials is metal dissolution before monomer oxidation, which prevents adherent and uniform film formation on the surface.^21,22^ To overcome this issue, metallic materials should be surface pretreated, which facilitates the electrodeposition process. Moreover, prior to utilizing any type of coating on Ti implants, it is vital to suitably pretreat the Ti alloy before the coating procedures. Chemical and mechanical treatment, degreasing, anodization, chemical brightening, powder coating and wet coating are the most available types of surface treatments for Ti alloys. In recent years, surface treatment has been used to create the necessary surface topography with morphological structures that further enhance the cellular response, including morphology, adhesion, proliferation, and differentiation.^23,24^ Therefore, surface pretreatment of the Ti alloy surface is likely a requisite; an optimum pretreatment should provide protection of the surface during electropolymerization, enabling good bonding to the surface.

In the past few decades, Ti-6Al-4V (G5) alloys have been extensively utilized as orthopedic implants; however, the dissolution of V and Al ions from Ti implants harshly disturbs the prolonged bioactivity of the Ti alloys due to the cytotoxicity and neurotoxicity of V and Al ions, which further impede mineralization of living bone. Hence, numerous novel Ti-based alloys have been fabricated for implant applications. Recently, the authors' research group has newly developed a nanograined Ti-20Nb-13Zr at% alloy with a near-β Ti microstructure from the nontoxic elements of Ti, Nb, and Zr,^25^ and the electrochemical corrosion and *in vitro* bioactivity of Ti–Nb–Zr alloys for orthopedic implant applications have been investigated.^26^ The developed alloy showed improved hardness and resistance to plastic deformation compared to commercial Ti and G5 alloys.^27^ To the best of our knowledge, there are no studies addressing the influence of surface treatment of Ti implants before electrodeposition of conductive polymer coatings for implant applications and a lack of comprehensive information about the surface features of Ti implants as a function of different surface treatment. Hence, the intention of this study is to find a suitable surface treatment for the newly developed Ti alloy substrates to sufficiently suppress the dissolution of Ti during the electrochemical deposition process and to thus offer temporary protection to facilitate a successfully electropolymerized PEDOT coating on the Ti alloy surface. Moreover, a suitable interfacial pretreatment layer between the Ti alloy substrate and the polymer coating would enable optimized corrosion and biological performance in physiological environments.

## 2 Experimental work

### 2.1 Materials and methods

The Ti alloy utilized in the present investigation is a newly developed near-β Ti-20Nb-13Zr at% (TNZ) alloy prepared through ball milling and spark plasma sintering techniques using elemental powders of Ti, Nb and Zr with 99.8% purity provided by Alfa Aesar, USA. TNZ alloy substrates 0.4 cm in thickness and 2 cm in diameter were ground using SiC grit sizes ranging from 320 to 2400 and lastly polished to a mirror-like surface. Subsequently, the substrates were ultrasonicated with acetone to eliminate residues and then dried in air. 3,4-Ethylenedioxythiophene (EDOT), lithium perchlorate (LiClO_4_), and acetonitrile (ACN) were procured from Sigma-Aldrich.

### 2.2 Surface treatment

Three different surface treatments were performed on TNZ substrates prior to electropolymerization. The first surface treatment (ST1) was carried out by dipping the substrates in a solution of 100 ml 48% H_2_SO_4_ and 18% HCl for 30 min, which was selected based on preliminary assessments. The second surface treatment (ST2) was carried out by immersion of the TNZ substrates in a mixture of HF : HNO_3_ : H_2_O at a ratio of 1 : 3 : 5 for 5 min at 60 °C. The third surface treatment (ST3) was performed by immersion of the TNZ substrates in 85% H_3_PO_4_ for 12 h at 60 °C. The TNZ substrates were cleaned with distilled water after surface treatment and dried in an oven.

### 2.3 Electrochemical synthesis of PEDOT coatings

The electropolymerization of EDOT on treated and untreated TNZ substrates was performed in a consistent three electrode cell assembly through cyclic voltammetric technique using an electrochemical workstation (Gamry Potentiostat Reference 3000, USA) in which the TNZ substrates acted as the working electrode, saturated calomel electrode (SCE) and graphite rod as the reference and counter electrode, respectively. Poly(3,4-ethylenedioxythiophene) was electrochemically synthesized on treated and untreated TNZ substrates by sweeping the potential between −0. 6 and 1.6 V at a scan rate of 0.05 V s^−1^ from a 0.3 M LiClO_4_/ACN solution containing 0.1 M EDOT monomer over 5 cycles. At the completion of the electrodeposition, coated TNZ substrates were removed from the electrolytic bath and washed with double distilled water to eliminate unreacted monomer molecules before being dried in air. The resultant PEDOT films were greenish black in color, compact, and very adherent to TNZ substrates. The thickness of the PEDOT coatings was measured using a conventional magnetic induction-based microprocessor-controlled coating thickness gauge (Elcometer Instruments, Germany) and the error in the thickness measurements was less than 5%. The average thickness of PEDOT coatings was found to be about 10–10.50 μm.

### 2.4 Surface characterization

The surface morphology and chemical composition were investigated using scanning electron microscopy (SEM, a JEOL microscope) with energy dispersive X-ray spectroscopy (EDS). Prior to analysis, TNZ substrates were treated with a thin platinum (Pt) film to diminish the influence of charging. For this purpose, the Pt film was deposited on TNZ substrates using the sputtering instrument (cressington sputter coater 108 auto, UK) and thickness of the film was maintained about 10 nm by controlling the deposition time. An optical profilometer (Contour GT-K, Bruker Nano GmBH, Germany) was used to measure the microscale roughness of the surface-treated and coated TNZ substrates. Three-dimensional images of the TNZ substrates were acquired by scanning an area of approximately 1.66 mm *×* 2.2 mm (3.5 mm^2^). Three images with a pixel resolution of 1632 × 786 were obtained from different locations on the TNZ substrates to calculate the surface roughness values. Attenuated total reflectance-infrared (ATR-IR) spectra of the synthesized PEDOT coatings were recorded in the range 400–4000 cm^−1^ by an IR reflectance spectrophotometry (Thermo scientific, with universal ATR attachment). The static and dynamic contact angles using sessile drop method were measured five times at different positions by a contact angle meter (VCA OPTIMA, AST Products Inc. USA), and the mean was selected as the final value. A drop of liquid (5 μl) was released onto the TNZ substrates and was imaged instantly after being located. The images of drops were analyzed using the image analysis system, which estimated the contact angles from the shapes of the drops with a precision of ±0.1°. Dynamic contact angles were measured using tilting cradle method (also referred to as the “inclined plate” method) and Fig. S1† shows the schematic representation of the static and dynamic mode (tilting cradle method).^28^ The liquid droplet is located on the substrate which is then gradually tilted. And a constant inclination angle was utilized for all the investigated TNZ substrates to eliminate the variances of conditions in the present study. Further, the surface free energy (SFE) of the TNZ substrates is calculated using the contact angles of two reference liquids (water and diiodomethane) through Owens Wendt Rabel Kaelble (OWRK) method.^29,30^ The model is based on the assumption that total SFE is the sum of the dispersion and polar components. While the polar part and the dispersive part of the surface tension of liquids are known, the SFE of a metallic substrates could be calculated by measuring the contact angle.

A CSM micro indenter and micro scratch machine (Micro Combi Tester CSM Instruments, Switzerland) was utilized for the scratch test using the standard Rockwell C indenter with a 100 μm tip radius. The indenter was pressed against the coating with an initial applied load of 30 mN and then pulled across the coating surface with progressive loading until the maximum applied load of 30 N was attained. The scratch test parameters utilized over a scanning length of 10 mm were a 5 N min^−1^ loading rate and a 10 mm min^−1^ scratch traverse speed, respectively. During the test, the normal load, penetration depth, acoustic emission (AE), frictional force and coefficient of friction (COF) were measured. By combining the friction curve and the acoustic emission signal, the critical loads *L*_c_ were determined, and this is the load at which adhesive failure of the coating occurs.

### 2.5 Electrochemical measurements

Electrochemical corrosion testing was accomplished in simulated body fluid (SBF) in a three-electrode typical electrochemical cell with a Gamry potentiostat/galvanostat controlled by Gamry framework software. The uncoated and coated TNZ substrates with an exposed area of 1.76 cm^2^ served as the working electrode. A graphite rod and a saturated calomel electrode (SCE) served as a counter electrode and a reference electrode, respectively. All potentials in the text refer to the SCE scale. After 1 h of stabilization at the open circuit potential (OCP), the linear polarization resistance (LPR) was recorded within the potential range of ±20 mV relative to the monitored OCP at a scan rate of 1 mV s^−1^. The electrochemical impedance spectrum was acquired in the frequency range from 10^5^ to 10^−2^ Hz in logarithmic increments with a 0.01 V amplitude using an alternating-current sine wave signal under potentiostatic conditions. The obtained impedance spectra were analyzed in terms of equivalent circuits using the in-built accessible software, and each experiment was repeated at least three times to confirm reproducibility.

### 2.6 *In vitro* cell culture studies

Human osteosarcoma cells (MG-63, Korean Cell Line Bank) were cultured in DMEM (GIBCO-BRL Rockville, MD) supplemented with 10% FBS, 1% penicillin and streptomycin in a cell incubator with 5% CO_2_ at 37 °C. MG-63 cells were trypsinized, 1 × 10^4^ MG-63 cells were seeded on UV-sterilized uncoated and coated TNZ substrates, and the plates were incubated in a 37 °C cell incubator. Assays were performed on days 5 and 7 using a live cell viability kit (Molecular Probes, USA). First, cells were washed twice with 1 × PBS to eliminate the non-adhered cells. Further, the live cells were stained with fluorescent dye (calcein AM at a working concentration of 2 μM in PBS). Plates were then incubated at room temperature in the dark for 20 min. Images of living cells (green-fluorescing cells) were visualized by fluorescence microscopy.

MG-63 cell suspensions containing 1 × 10^4^ cells were added to 6-well plates containing uncoated and coated TNZ substrates. Cells were allowed to grow on the specimens for 5 and 7 days and then transferred to new 6-well plates, after which an assay was performed as per the Cell Counting Kit-8 (CCK-8) protocol (Dojindo, Molecular Technologies, Rockville, MD, USA). Absorbance was measured at 450 nm using a spectrophotometer.

## 3 Results and discussions

### 3.1 Surface characterization of surface-treated TNZ alloy

**Fig. 1:**
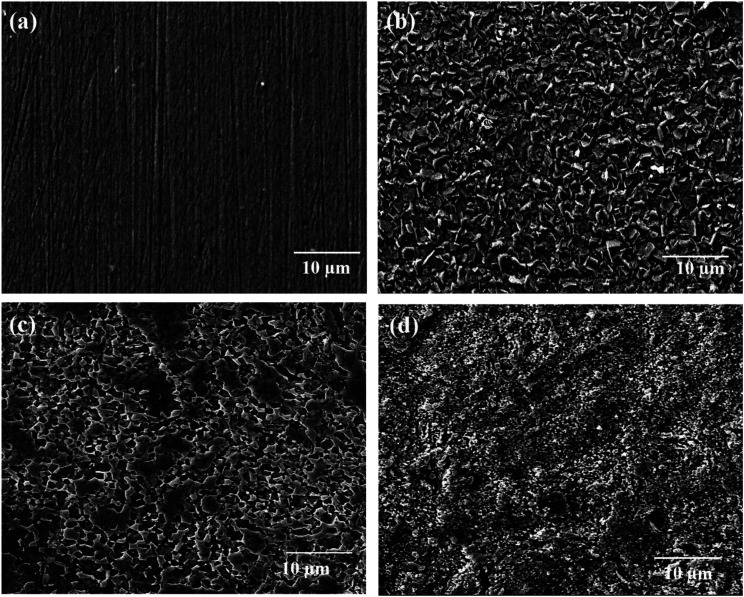
SEM images of (a) untreated TNZ, (b) ST1, (c) ST2 and (d) ST3 TNZ substrates.

Fig. 1(a–d) display the SEM images of untreated and treated TNZ substrates. The SEM images of UT TNZ substrates exhibited a flat surface, smoothly scratched with unidirectional grooves and tiny scratches left during the grinding operation. Conversely, after surface treatment, the surface microstructure was completely transformed. A flake-like structure was observed in moderately flat regions for ST1, which is depicted in Fig. 1b; the surface is homogenous, in which peaks and sharp edges are clearly observed. The SEM image of ST2 exhibited a lamellar microstructure containing clear separation of α and β phases, which was certainly detected by the segregation of brighter (β-Ti-bcc matrix) and darker (α-Ti-hcp) areas.^25^ In the case of ST3, an island-like surface morphology with indiscriminately dispersed precipitates was observed on the surface along with a few micropores. ST3 exhibits a surface morphology with micro-rough surface structures. Chen *et al.* also electrochemically etched the Ti implant surface and attained the roughest surface with big pits and cavities which were reasonably appropriate for the growth of bone cells, tissue and bone fixity.^31^ Schliephake *et al.* reported that the acid etched Ti implant surfaces possibly reduce the abrasion from fixture surfaces and provide a biologically favorable surface structure.^32^ EDS analyses of untreated and treated TNZ substrates are displayed in Fig. S2 as ESI.† The absence of elements other than Ti, Nb and Zr in the EDS results of the treated TNZ surface demonstrated that the surface treatment probably removes the oxide layer and the impurities on the surface of the TNZ substrates.

**Fig. 2:**
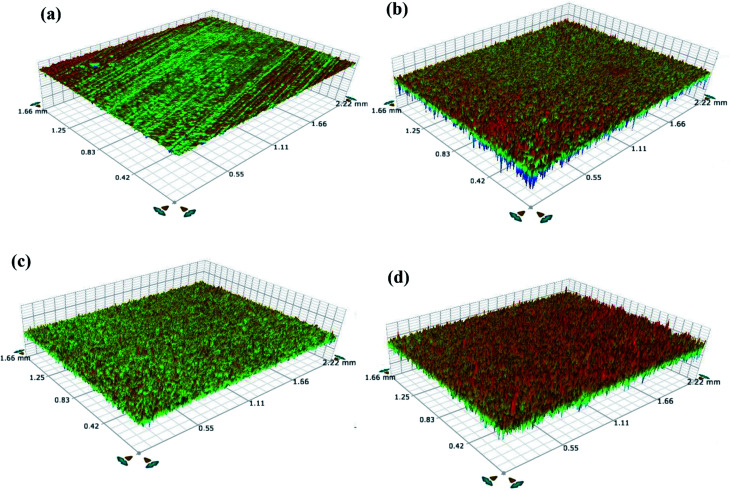
Surface topographic images of (a) untreated TNZ, (b) ST1, (c) ST2 and (d) ST3 TNZ substrates.

Fig. 2(a–d) display optical profilometry topography images for the treated and untreated TNZ substrate surfaces. The untreated TNZ surface exhibits a smooth surface with no evident topographies, whereas the treated TNZ surfaces display rough surfaces. From these images, it is evident that the roughness of the untreated TNZ surface is lower than that of the treated TNZ surfaces. The substrate roughness is also a significant factor during the electrodeposition of polymer on a substrate surface. The quantification of “surface roughness” can be performed using a selection of different parameters, such as the root mean square height of the surface (*R*_q_), maximum height of peaks (*R*_p_), maximum depth of valleys (*R*_v_), ten-point height (*R*_z_), and arithmetic average height of the surface (*R*_a_). In general, *R*_a_ (average surface roughness) and *R*_q_ (root mean square, RMS) are important factors for analyzing surface roughness because they both provide an idea about the frequency of deviations from a smooth surface by examining a continuous surface profile. In the present study, the values of *R*_a_ and *R*_q_ for the untreated TNZ substrates (Table 1) were found to be low (between 0.173 and 0.26 μm) and were increased to 3–20 μm for treated TNZ substrates. In addition, *R*_z_ values are generally measured based on the five highest peaks and the five deepest valleys in relation to a straight centerline. Hence, this approach offers evidence on the extremes of the surface heterogeneity/irregularities.^33^ Comparing the *R*_z_ values of treated and untreated surfaces, the results indicated that the *R*_z_ value increased with the surface treatment, and the maximum value was obtained for ST3. The result revealed a significant increase in the mean surface roughness after surface treatments, and particularly, the highest roughness parameters belonged to the ST3 TNZ sample. Stango *et al.* have investigated the effect of laser texturing on the adhesion strength of hydroxyapatite coatings on Ti-6Al-4V implant and found that increased surface roughness result in the easier incursion and lead to better grip to hold the other molecules on the Ti implant surface.^34^ Further, Buser *et al.* reported that the surface roughness acquired after surface treatment improve the osteoconductive phenomenon, bone to implant interaction and increase removal torque.^35^ An increase in surface roughness generally facilitates the creation of physical interactions of polymer molecules with the Ti surface and thus increases adhesion through mechanical interlocking theory.

**Table 1:** Surface roughness parameters of TNZ substrates

Substrates	*R*_a_ (μm)	*R*_p_ (μm)	*R*_q_ (μm)	*R*_z_ (μm)	*R*_v_ (μm)
UT	0.173	1.824	0.26	12.11	−10.287
ST1	3.123	29.01	4.32	72.77	−43.68
ST2	7.19	72.37	9.60	143.03	−70.65
ST3	15.74	61.39	20.11	193.68	−132.29
PEDOT/UT	14.93	91.79	18.76	175.01	−83.29
PEDOT/ST1	15.35	94.52	19.26	193.40	−98.87
PEDOT/ST2	15.10	82.71	18.95	165.35	−82.64
PEDOT/ST3	15.98	101.01	20.10	196.6	−95.58

**Fig. 3:**
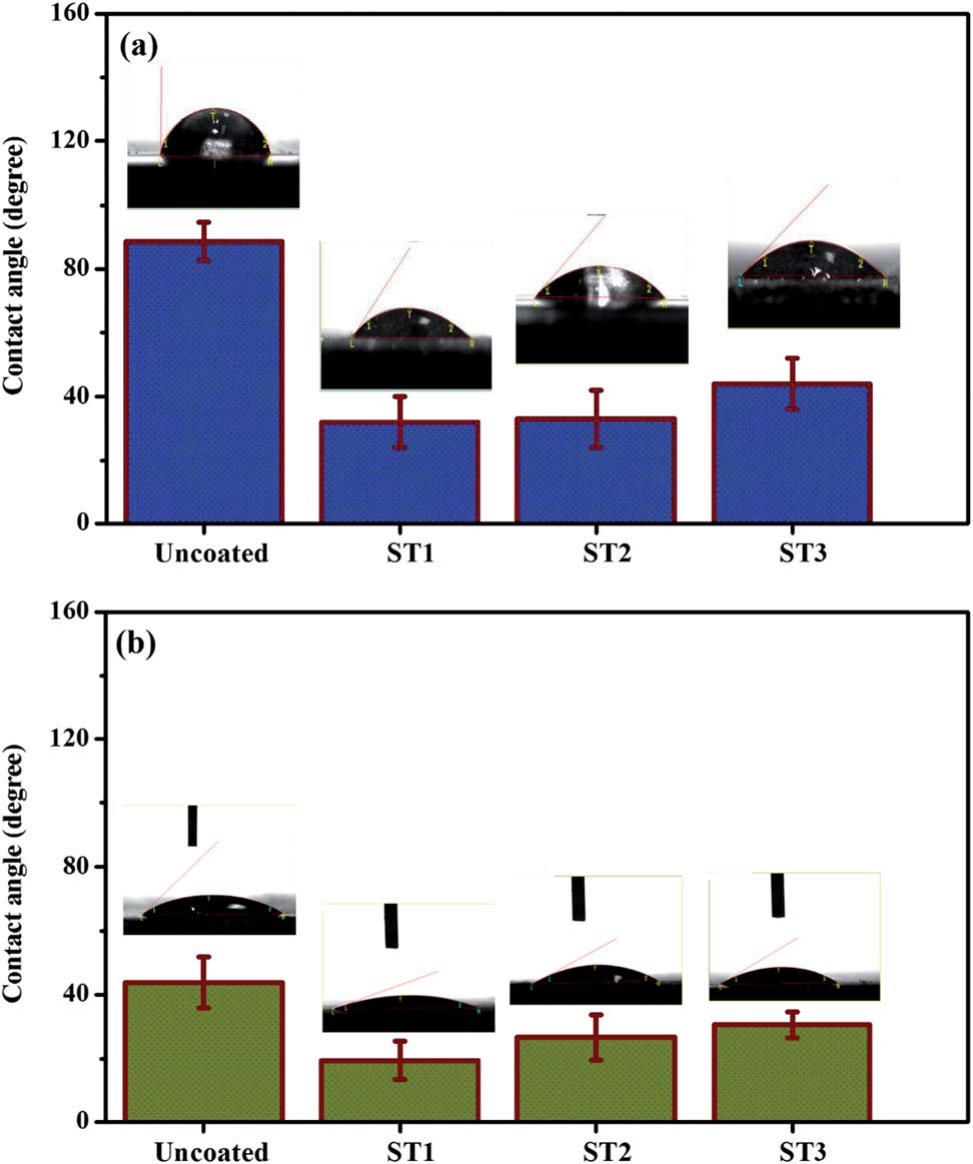
Contact angle results of (a) water and (b) diiodomethane for untreated and treated TNZ substrates.

Exploring the wettability of the surface is one of the main accounts to govern the appropriateness of the surface for bonding with others materials. In general, the change in wettability is strongly associated to the surface energy variation and generally a material surface with high surface free energy leads to an enhanced wetting. The influence of surface treatment on the surface wettability of the TNZ samples was investigated by measuring the contact angles of two different liquids (water and diiodomethane) and the results are shown in Fig. 3(a and b). Untreated TNZ substrates showed a water contact angle (WCA) of 88.50°, which reveals the hydrophobicity of the untreated TNZ surface due to the existence of an oxide film on its surface. In contrast, the WCA of TNZ substrates after surface treatment was considerably decreased (approximately 30°), which could be associated with the increase of surface roughness and the removal of the oxide layer after surface treatment. Bathomarco *et al.* established that the measured contact angle decreases with increasing the surface area of titanium implants.^36^

The surface free energy (SFE) of a material surface provides a significant information about the intermolecular interactions at interfaces and exhibit a strong impact on wetting, adsorption and adhesion behavior with other materials. SFE and wettability of materials can be determined by measuring the contact angle formed by a range of liquids on a given surface, using several different approaches. In the present study, the SFE of the untreated and treated TNZ substrates was calculated using contact angles and the variation of the dispersive and polar components of surface free energy and the total SFE were summarized in the Table 2. The most hydrophobic nature and the minimal values of SFE belong to untreated TNZ surface. The obtained results demonstrate that the SFE calculated by the OWRK method is found to be in the range of 65–90 mN m^−1^ and their polar parts are in the range of 20–40 mN m^−1^ for treated TNZ surfaces. Gentleman *et al.* designated the SFE as a necessary feature of the material surface which primarily govern the first interactions with the biological surroundings.^38^ Kilpadi *et al.* reported that acid etching samples exhibited higher SFE, which advances the interaction between implant and surrounding bone.^39^ The increase in SFE is attributed to the increase of surface roughness and also the removal of oxide layer from the TNZ surface after surface treatment.

**Table 2:** Contact angle and surface free energies of untreated and treated TNZ substrates

Substrates	Contact angle (°)		Surface free energy (mN m^−1^)		
	Water	Diiodomethane	Polar	Dispersive	Total
UT	88.7 ± 4	43.8 ± 6	1.1	28.6	29.7
ST1	32 ± 5	19.4 ± 4	39.7	49.4	89.1
ST2	33 ± 6	26.6 ± 5	36.5	46.3	82.8
ST3	44 ± 5	30.5 ± 4	23.9	41.6	65.5

Measuring the static contact angle is a common approach to investigate the wettability of material. However, it is considered to be limited to provide the information about the surface chemistry and hydrophilicity of materials surface as it does not consider the relative movement of the various interfaces. Thus, dynamic contact angles are also be measured in the present study to get more insight on wettability influenced by surface chemistry. In general, contact angle hysteresis (CAH = cos *θ*_adv_ − cos *θ*_rec_) is the difference between the advancing and receding contact angles. The importance of CAH has been effectively studied by several researchers,^40,41^ and the common conclusion is that it mainly arises from the chemical and topographical heterogeneity, surface deformation, and adsorption and desorption, swelling and penetration.^42^ Dynamic contact angle images of untreated and treated TNZ surface were measured and the images were presented as ESI in Fig. S3.† As anticipated, untreated TNZ surface showed the lowest wettability followed by ST3, ST2 and ST1 TNZ surfaces. An increase in CAH due to shifts of the receding CA only was detected with untreated TNZ surface, demonstrating a sensitivity of advancing and receding CAs due to the presence of oxide layer and thus, an increase in CAH is ascribed to the surface heterogeneity of untreated TNZ substrates.^43^ Besides, a decrease in CAH is initiated by surface treatment due to the removal of oxide layer over TNZ surface. It has been already reported that the microstructured sandblasted and acid-etched Ti alloy surfaces with increased hydrophilicity exhibit no apparent hysteresis.^44^ Furthermore, Rupp *et al.* reported values of water receding angles equal to zero for acid-etched titanium surfaces.^45^ The obtained results implied that the surface treatment of the TNZ substrates could efficiently improve its hydrophilicity and surface free energy, which is a favorable state for the electrochemical synthesis of polymer coatings.^37^

### 3.2 Electrochemical synthesis of PEDOT films on treated TNZ substrates

**Fig. 4:**
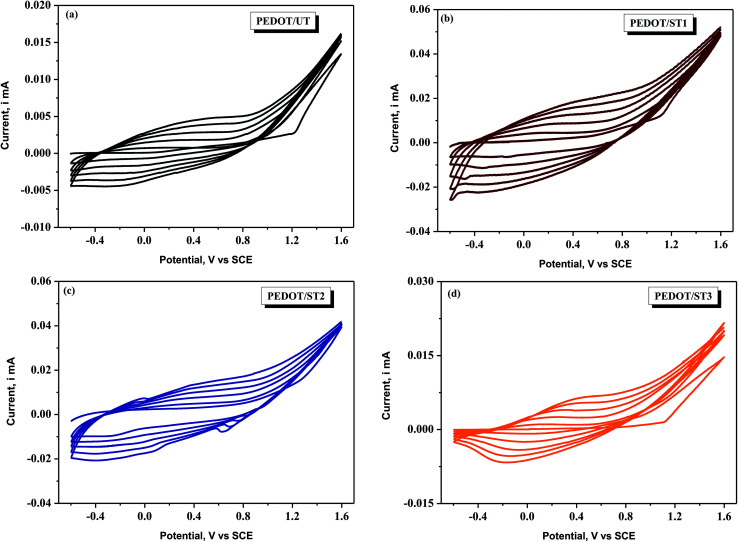
CV curves of PEDOT coatings synthesized on untreated and treated TNZ substrates by sweeping the potential between −0. 6 and 1.6 V at a scan rate of 0.05 V s^−1^.

Fig. 4(a–d) display the CV curves for the electrochemical synthesis of PEDOT coatings on treated and untreated TNZ substrates from a solution of 0.1 M EDOT in a solution of 0.3 M LiClO_4_/ACN. In the first forward scan, the oxidation of EDOT occurs at approximately 0.90 V (*vs.* SCE) with a significant rise in the current related to the formation of EDOT radicals from the EDOT monomer. In the inverse scan, the cathodic peak was not detected, confirming the irreversible oxidation of the monomer. In consecutive cycles, the current increased progressively and a current loop arisen between 0.90 V and 1.6 V, demonstrating the formation of the PEDOT film.^46^ Gopi *et al.* also found the similar results and they mentioned that the increase in current at the end of each cycle could be ascribed to the process of EDOT oxidation, which occurs in a step by step manner leading to the formation of the PEDOT film with increasing thickness.^47^ By comparing the CV curves of PEDOT coatings deposited on treated and untreated TNZ substrates, it can be observed that monomer oxidation occurs at the same potential. However, the current associated with the formation of PEDOT is higher in the case of PEDOT on treated TNZ substrates, indicating that the modified TNZ surface has an influence on the formation of PEDOT coatings. Visual examination during CV experiments indicated the formation of a compact and homogeneous PEDOT film greenish black in color on TNZ substrates.

### 3.3 Surface characterization of PEDOT films synthesized on treated TNZ substrates

**Fig. 5:**
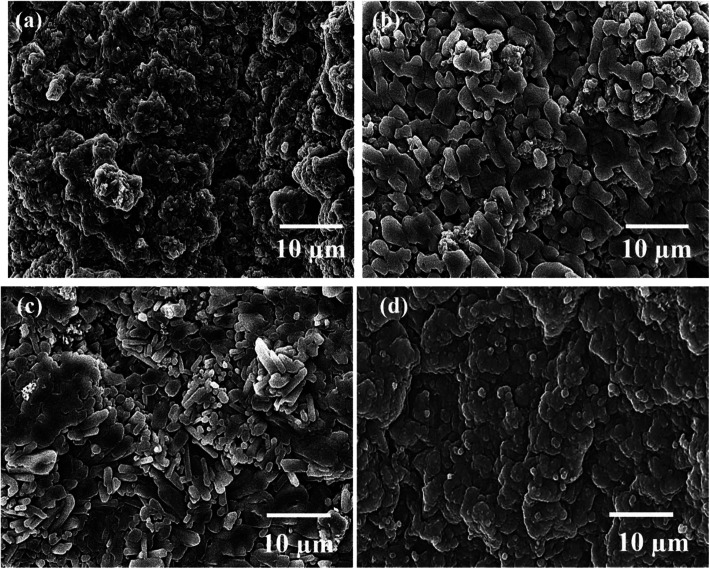
SEM images of (a) PEDOT/UT, (b) PEDOT/ST1, (c) PEDOT/ST2 and (d) PEDOT/ST3 TNZ substrates.

Fig. 5(a–d) present the surface morphologies of PEDOT coatings synthesized on untreated and treated TNZ substrates. The morphology of PEDOT coatings on untreated TNZ substrates was a well-developed granular morphology formed by the accumulation of globular grains with irregular void spaces in between. SEM micrographs of PEDOT coatings on treated TNZ substrates indicated a slight alteration with varying surface treatment. As depicted in Fig. 5b, the PEDOT/ST1 substrates revealed a more compact morphology with a uniform arrangement of the granular structure without any cracks in between. In contrast, PEDOT/ST2 substrates displayed uniform elliptical rods with a diameter of approximately 1 μm, which might be related to the polymerization reactions that occurred at the treated TNZ surface. Furthermore, PEDOT/ST3 substrates presented a less compact and asymmetrical granulated arrangement with fewer pores and few cracks in between. Castagnola *et al.* investigated the influence of three different electrochemical methods on nucleation mechanism of PEDOT electropolymerization and found the different surface morphology and topography which is explained by the base substrate effect on the starting growth process.^48^ Patra *et al.* also prepared the PEDOT coatings on SS substrate by galvanostatic (GS), potentiostatic (PS) and potentiodynamic (PD) methods and compared the surface morphology of synthesized coatings. Their results concluded that the PEDOT coatings synthesized at low current densities and potentials delivered the globular surface morphology and the morphology turns out to be porous at higher current densities and higher potentials of preparation. In the case of PD route, however, the morphology becomes rod-like and fibrous.^49^ It is believed that the treated TNZ surface stimulated all the initiator monomers to form a PEDOT matrix, which represented the template for the 1D growth of the monomer and inhibited the subordinate growth of PEDOT. In addition, EDS analysis was performed on the coated TNZ substrates, and the results are presented in Fig. S4 as ESI.† The appearance of C, O and S peaks from EDOT molecules and further the presence of chlorine peaks arisen due to the supporting electrolyte (LiClO_4_) and the absence of peaks from base substrates further confirmed the continuous and compact PEDOT layer on TNZ substrates.

**Fig. 6:**
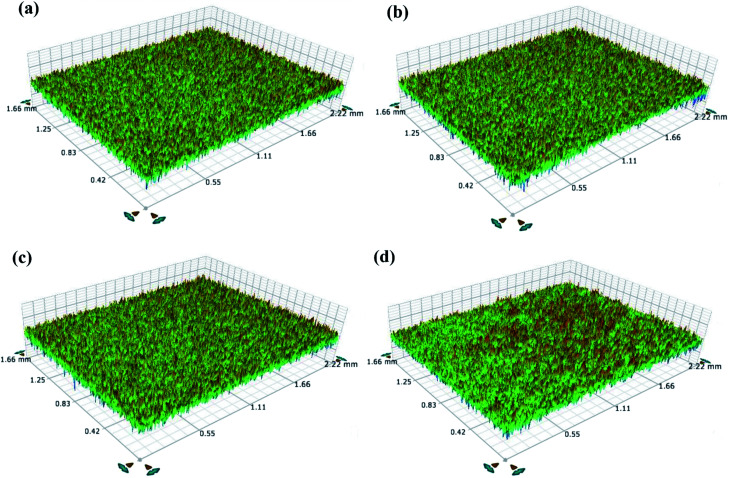
Surface topographic images of (a) PEDOT/UT, (b) PEDOT/ST1, (c) PEDOT/ST2 and (d) PEDOT/ST3 surfaces.

Surface topographic images of synthesized PEDOT coatings on treated and untreated TNZ substrates are displayed in Fig. 6(a–d), and the measured roughness values are listed in Table 1. PEDOT coatings on treated TNZ substrates exhibited a compact, relatively smooth surface with visually uniform peaks. PEDOT/ST substrates displayed a perfectly random porous texture with predominant valleys. Numerous investigations^50–52^ assessed the favorable outcome of rough surface acquired by surface treated practice on osseointegration process. In general, the surface roughness of the polymer coating is a significant feature that can obviously influence its corrosion protection behavior and further properties, including hydrophobicity, appearance and bioactivity in physiological environments.

### 3.4 Structural characterization of PEDOT films synthesized on treated TNZ substrates

**Fig. 7:**
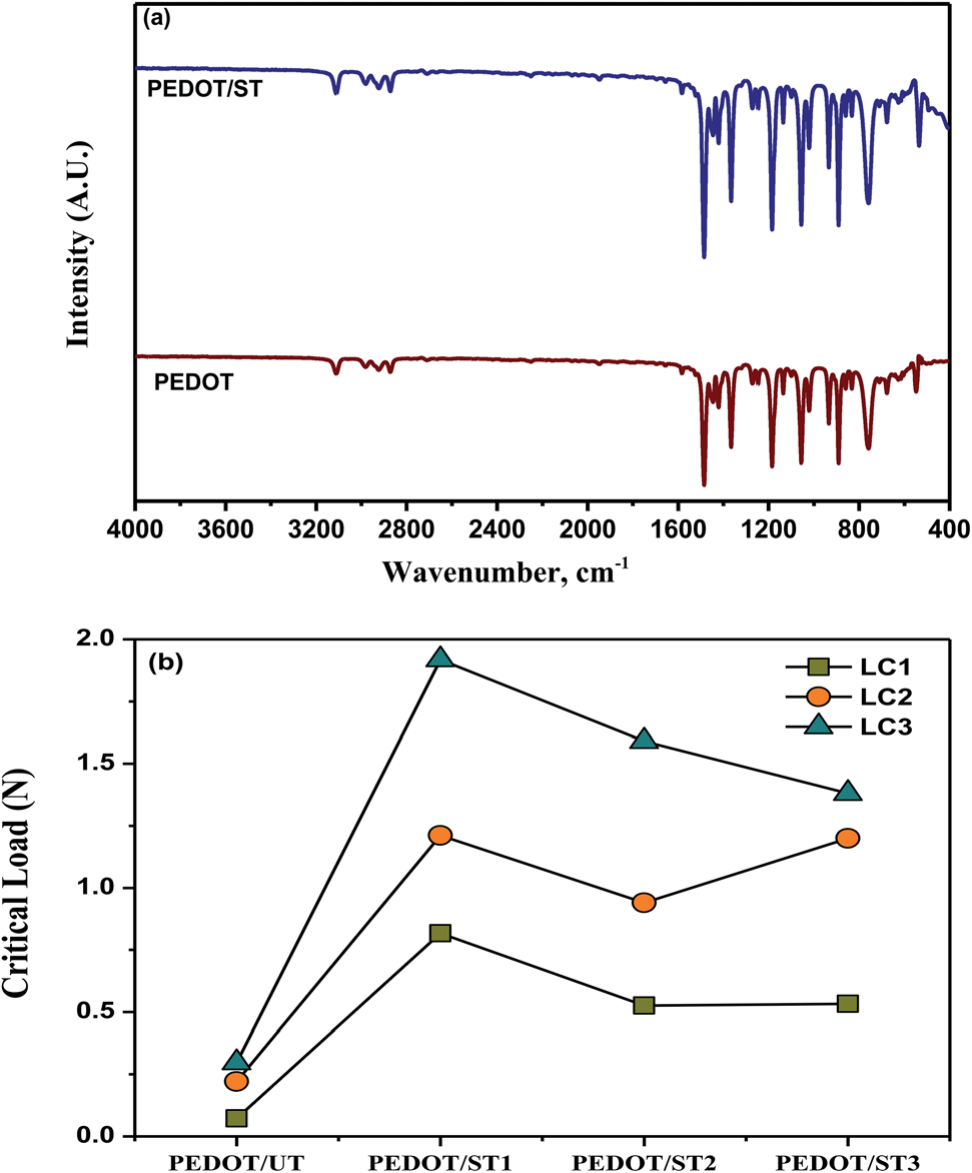
(a) IR curves of PEDOT coatings, and (b) scratch resistance results of PEDOT coatings on treated and untreated TNZ substrates (*L*_c1_ – first critical load, *L*_c2_ – second critical load and *L*_c3_ – third critical load).

The ATR-IR spectra of PEDOT coatings deposited on untreated and treated TNZ substrates are shown in Fig. 7a. For the IR spectra of PEDOT, the vibrational peaks at about 1500 and 1365 cm^−1^ were ascribed to the C=C and C–C stretching vibrations of the quininoid structure of the thiophene ring, respectively. The obtained peaks at about 1195, 1137, and 1062 cm^−1^ were arisen due to the C–O–C bond stretching modes in the alkylenedioxy group. Moreover, the C–S bond in the thiophene ring was verified by the existence of peaks at about 932 and 892 cm^−1^. The sequence of peaks recommended the formation of PEDOT on TNZ substrates.^19^ The obtained results are well agreement with the literatures.^18,19^ By comparing the spectra of PEDOT deposited on untreated and treated TNZ substrates, it can be clearly revealed that both spectra are very similar, which indicates that the chemical structure of the deposited films on both substrates is the same.

### 3.5 Adhesion test of the PEDOT coatings on untreated and treated TNZ substrates

The high adhesive strength of coatings to metallic implants is one of the basic desires to utilize the coated implants in orthopedic applications. Scratch resistant test is a beneficial practice to estimate the nature of adhesion strength of the coatings on an implant surface and the disinfected loosening of implant, delamination of coating during implantation, and allergies due to wear debris are main concerns.^53^ The adhesion of polymer coatings on metallic substrates is generally evaluated through scratch resistance by describing the critical loads and evaluating their stage alongside the scratch profile. The appearance of a bright surface on the scratch profile is a sign of delamination, and the corresponding load is denoted as a critical load (*L*_c_). In general, three different critical loads are obtained during scratch resistant analysis.^54^ The first critical load *L*_c1_ represents the applied normal load at which the initial noteworthy tears appear alongside the scratch profile, and the surface beneath typically comes into focus. The second critical load *L*_c2_ relates to the applied normal load at which the propagation of fracture occurs, therefore linking both the bottom and the edge of the scratch profile. Finally, the third critical load *L*_c3_ represents the applied normal load at which the coating displays a terrible failure, with partial or complete coating delamination. It has been already reported that *L*_c_ representing its resistance to scratch and higher load carrying capacity can be influenced by the coating's thickness, phase composition, hardness and its porosity.^55^

**Fig. 8:**
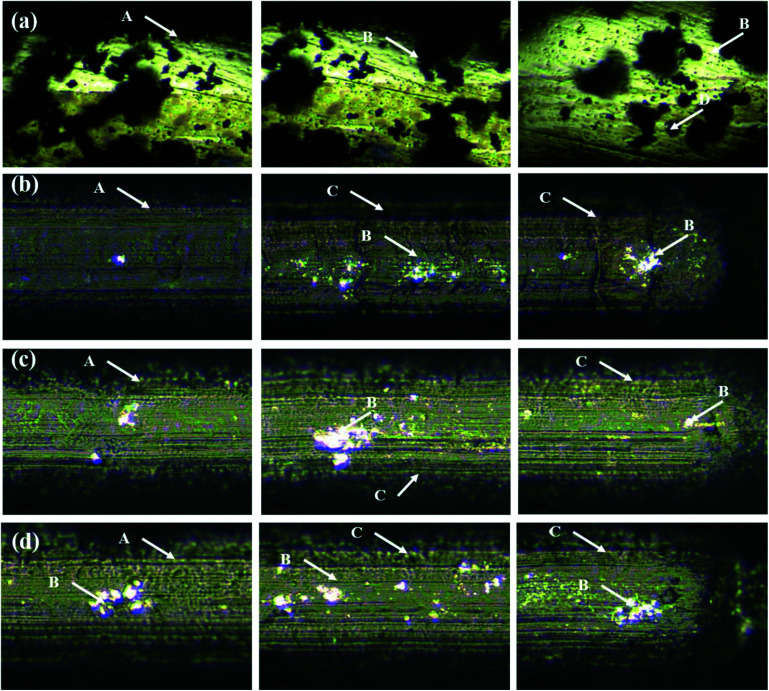
Optical images of scratch profiles of (a) PEDOT/UT, (b) PEDOT/ST1, (c) PEDOT/ST2 and (d) PEDOT/ST3 coatings.

The obtained *L*_c_ values for the PEDOT coatings on treated and untreated TNZ substrates are summarized in Fig. 7b, and respective optical images of scratch profiles are shown in Fig. 8. The sites of coating spallation (A), delamination (B), brittle chipping (C) and gross spallation (D) are clearly noticeable in the obtained optical images.^56^ The adhesion of the PEDOT coating to the untreated TNZ substrate was lower, as it experienced cohesive failure at the critical load of 0.07 N. Further, the second critical load was observed at 0.22 N, in which continuous tearing and detachment of the coating from the substrate occurred. In contrast, the higher adhesion strength of PEDOT coatings on treated TNZ substrates compared to the untreated surface was indicated by the higher critical load of approximately 2 N. In particular, PEDOT/ST1 substrates presented the maximum *L*_c_ values among the investigated coatings, representing the improved performance of adhesion of polymer coatings to the treated TNZ surface. Cao *et al.* investigated the scratch resistance of magnetron sputtered TiAlSiN coatings and reported that the critical load values are directly be governed by coatings' elastic and plastic deformation.^57^ Further, Laouamri *et al.* have also investigated the effect of the acid etching on the scratch resistance and interfacial adhesion of acrylic-coatings on sandblasted glasses and established that scratch resistance of acrylic coatings is higher in the case of acid etched sandblasted samples due to the good mechanical interlocking at the interface promoted by the micro-corrugation.^58^ Consequently, the coating becomes more able to withstand external loading circumstances as the existence of internal stresses is diminished by the corrugated morphological features of the substrate surface itself.^59^ In the present study, the obtained scratch-resistant results emphasize the beneficial impact of surface treatment on the adhesion strength of the PEDOT coatings on TNZ surfaces. From the surface analysis findings, it was already concluded that the nature of the PEDOT coating (surface morphology, topography and hydrophobicity) was intensely altered by the surface treatment performed before electrodeposition, which could play a favorable role in the adhesion strength of the polymer coating because the surface area of the treated TNZ interface might become larger during the surface treatment, hence increasing the adhesion strength. Additionally, the surface treatment removed the oxide layer and hence improved the surface free energy of the substrate, which improved adhesion with the coating. Moreover, the surface treatment increased the surface roughness, which enhanced the adhesion of the coating through mechanical interlocking.

### 3.6 Electrochemical corrosion studies of PEDOT coatings on treated TNZ substrates

**Fig. 9:**
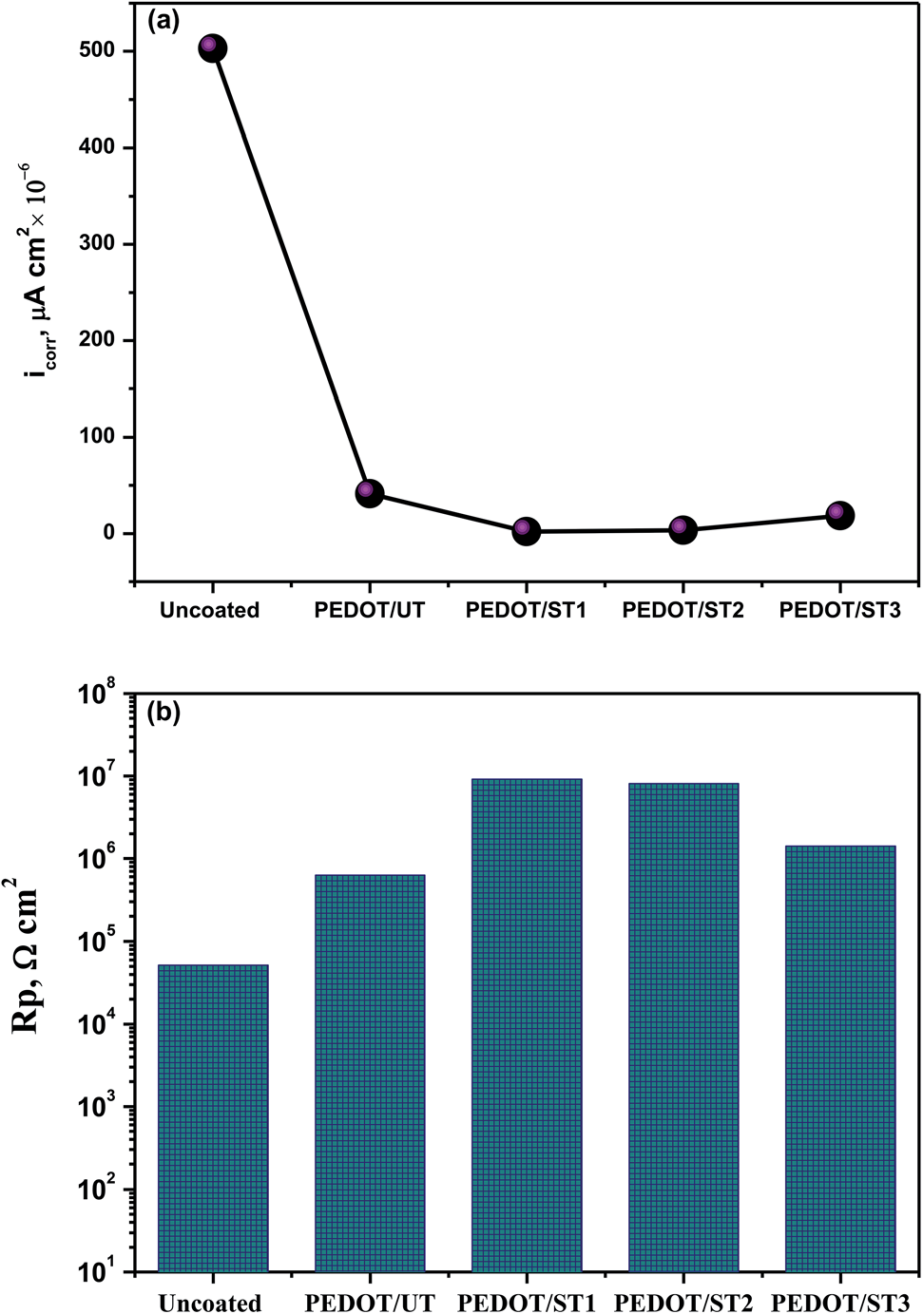
(a) *i*_corr_ and (b) LPR values of PEDOT coatings on untreated and treated TNZ substrates in SBF medium.

Linear polarization results for the uncoated and coated TNZ substrates in SBF medium are presented as ESI in Fig. S5.† The obtained *i*_corr_ and polarization resistance (*R*_p_) values are displayed in Fig. 9(a and b), providing a direct indication of the coating stability during immersion in SBF medium. Uncoated TNZ substrates showed an *i*_corr_ value of 503 × 10^−9^ A cm^−2^, and the presence of the PEDOT coating strongly decreased the current density, which indicated the lower corrosion rate of coated TNZ substrates. In particular, the *i*_corr_ value of the PEDOT coatings on treated TNZ substrates, especially PEDOT/ST1 (1.852 × 10^−9^ A cm^−2^), was significantly reduced compared to PEDOT/UT (41.240 × 10^−9^ A cm^−2^), highlighting the critical role of surface treatment prior to electrodeposition to improve the corrosion protection performance of polymer coatings. Ogawa *et al.* reported that acid etching process effectively alter the Ti surface, due to its enhanced corrosion protection performance in all electrolytes assessed as well as good surface features after the corrosion process and their results also delivered significant information about the higher *in vivo* success rate of dental implants treated with acid etching when compared to others.^60^ Further, the calculated *R*_p_ for the PEDOT coatings on the untreated TNZ surface was found to be 6.318 × 10^5^ Ω cm^2^, while for the PEDOT/ST substrates, it was found to be in the range of 10^6^ Ω cm^2^. In particular, PEDOT/ST1 substrates exhibited the highest *R*_p_ value, which further confirmed the enhanced barrier performance of PEDOT coatings prepared on the treated TNZ surface. Based on the obtained results from LPR, it can be preliminarily determined that the surface treatment improved the performance of PEDOT coatings, which was probably due to the deposition of a more compact and adhesive layer of PEDOT coating on treated TNZ compared to the untreated surface.

**Fig. 10:**
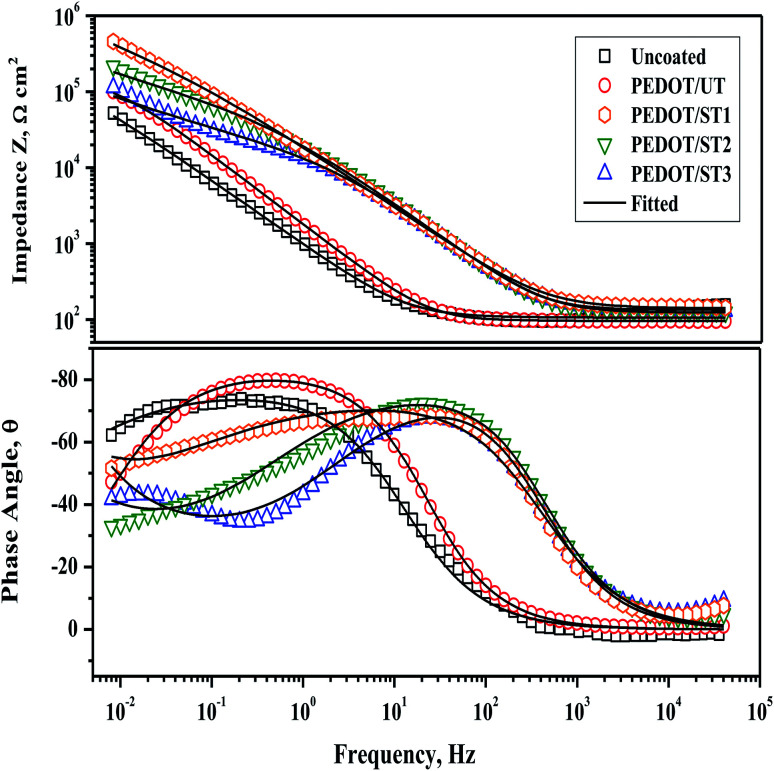
EIS curves of PEDOT coatings on untreated and treated TNZ substrates in SBF medium.

Further, EIS analysis of the uncoated and coated TNZ substrates was performed to obtain more information, including the dielectric behavior of the polymer coatings and diffusion and electrochemical reactions occurring at the metal/coating interface. The EIS results for coated and uncoated TNZ substrates in SBF medium are presented in the Bode format in Fig. 10. The obtained experimental data are expressed as symbolic dots, whereas simulated curves are presented as solid lines. From a closer observation of the Bode plots, particularly the phase angle curves, it is clear that the coated TNZ substrates exhibited nearly one extensive range time constant comprising two uncertain arcs, which was probably caused by the limited corrosion resistance of the polymer layer due to its pores and cracks. The first time constant appearing at the higher frequency of 1–10 Hz is linked to the polymer layer, and the second time constant at the lower frequency of approximately 1 mHz is associated with charge transfer reactions at the metal/coating interface through the pores of the polymer coating.^61^ From the Bode plots, the impedance modulus (*Z*) of PEDOT/ST substrates is higher than that of PEDOT/UT and uncoated substrates, which might further highlight the efficient role of surface treatment on the TNZ substrates before electrodeposition.

Interpretation of the EIS curves of the investigated substrates was made by fitting the curves with a suitable equivalent electrical circuit, as displayed in Fig. S6 as ESI,† where *R*_s_ denotes the solution resistance, *R*_ct_ means the charge transfer resistance, *R*_f_ is the film resistance and CPE_dl_ and CPE_f_ represent the double-layer capacitance and coating capacitance of the constant phase element (CPE). The CPE is employed as an alternative for a pure capacitance because perfect capacitive performance cannot be obtained in actual electrolytes. Moreover, the usage of CPE reduces the error and offers more comprehensive evidence about the non-ideal dielectric aspects of the polymer coating. The impedance of the CPE mentioned is given by the following relation:

1 *Z*_CPE_ = 1/[*Y*_0_(*jω*)^*n*^]

where *Y*_0_ is the admittance of the CPE, *ω* represents the angular frequency and *n* denotes the dispersion exponent. When *n* = 1, CPE signifies a pure capacitance, and when *n* = 0, CPE becomes an ideal resistor.^62,63^ The electrochemical parameters acquired from the EIS fitting are listed in Table 3. Compared with the *R*_ct_ of the uncoated substrate, there is a noteworthy increase in *R*_ct_ for the TNZ substrates coated with PEDOT, representing that the electrodeposited polymer films could efficiently offer barrier protection to underlying TNZ substrates. Moreover, the changes in the *R*_ct_ values could be described by the relative area of the metal/solution interface, which was largest in the case of the uncoated substrate, as the resistance was lower, and decreased for the polymer-coated substrates.^64^ In particular, PEDOT/ST1 coatings exhibited the highest *R*_ct_ value, which further highlights the synergetic effect of surface treatment before electrodeposition. A lower CPE_f_ represents a lower porosity of the coatings, and the low CPE_f_ value for PEDOT/ST1 designates its lower porosity (compact and dense barrier layer), which provides its higher corrosion resistance in SBF.^65^ In addition, PEDOT/ST1 exhibited a reduction in CPE_dl_ by one order of magnitude, which implies an effective decrease in the solution-exposed surface and thus a high degree of coating coverage. Evaluation of the corrosion protection performance of polymer coatings is generally governed by parameters such as *R*_f_ and *R*_ct_; *R*_f_ specifies the resistance of a coating against diffusion of aggressive species from the electrolyte, and *R*_ct_ denotes the resistance to electron transfer across a metal/coating interface. Accordingly, the *R*_f_ and *R*_ct_ of PEDOT coatings are more prominently enhanced when the coatings are deposited on the treated TNZ surface. The obtained EIS results further corroborate that the PEDOT coatings prepared on treated TNZ surfaces, displayed more adherent and shielding films on the surface-treated TNZ substrates. During the electrodeposition of PEDOT coatings, the EDOT molecules systematically formed a compact and defect-free polymer layer on the surface-treated substrates, thus resulting in the formation of well-adherent coatings on the TNZ surface.

**Table 3:** EIS parameters for uncoated and coated TNZ substrates

Substrate	*R*_s_, Ω cm^2^	*R*_ct_, kΩ cm^2^	*Q*_dl_, μF cm^−2^	*n*_dl_	*R*_f_, kΩ cm^2^	*Q*_f_, μF cm^−2^	*n*_f_
Uncoated	125	50.14	62.28	0.85	—	—	—
PEDOT/UT	134	92.12	20.74	0.92	2.10	11.25	0.94
PEDOT/ST1	129	386.91	0.13	0.98	22.32	0.85	0.97
PEDOT/ST2	114	194.28	0.58	0.96	17.89	0.92	0.96
PEDOT/ST3	123	121.52	0.92	0.95	11.54	1.28	0.94

### 3.7 *In vitro* bioactivity studies

**Fig. 11:**
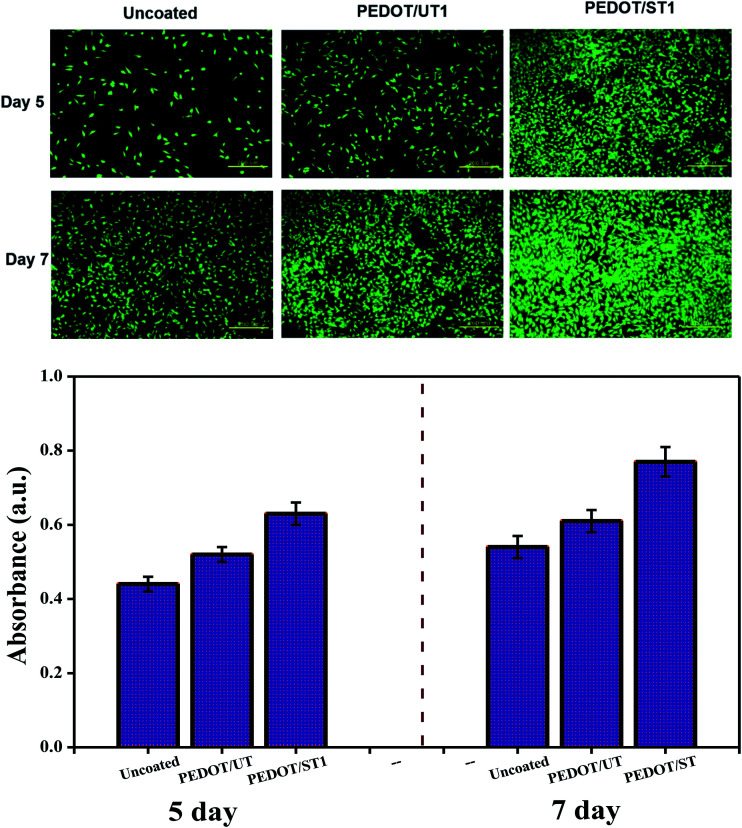
*In vitro* MG63 osteoblast cell culture studies of PEDOT coatings on untreated and treated TNZ substrates.

Fig. 11(a and b) display the cell proliferation and morphology on the uncoated and coated TNZ substrates after 5 and 7 days of culture. The cells were well grown, displaying a polygonal shape with filopodial extensions, and they spread entirely on the coated TNZ substrates, in contrast to the uncoated substrates. In general, osteoblast growth in material-free organ culture can be categorized into four stages, *viz.* cell adhesion, attachment, spreading and proliferation.^66^ After cell attachment, the osteoblasts initiated a period of prompt proliferation, which was confirmed by the difference in the cell number between 5 and 7 days on the uncoated and coated TNZ substrates. On uncoated TNZ substrates after 7 days, the cell density appeared to be lower compared to coated substrates. On day 7, cells cultured on PEDOT coatings on treated TNZ substrates (Fig. S7†) were well spread with a polygonal shape and spread uniformly across the coating on the treated TNZ substrates.

Fig. 11b presents the number of MG63 cells after proliferation for 5 and 7 days on the surfaces of uncoated and coated TNZ substrates. After 5 days of culture, the cell number displayed no significant difference between the uncoated and PEDOT/UT coated substrates, which was in agreement with the cell adhesion results. After 7 days, the cell numbers on uncoated substrates were significantly lower (0.55) than those on coated substrates, which indicates that the PEDOT coatings had higher cytocompatibility than the uncoated TNZ substrates. In particular, the proliferation rate on PEDOT/ST was much higher than that on PEDOT/UT and uncoated substrates after culturing for 5 days. Further, the number of cells on PEDOT/ST continued to grow prominently after culturing for 7 days, while the proliferation rate on the PEDOT/UT and uncoated substrates was very low. Consistent results were achieved by other researchers and it has been already reported that surface roughness, topography at the micro- and nano-scale could impact cell morphology and proliferation.^67,68^ Altankov *et al.* have also described that increased cell proliferation has been showed in materials with greater surface wettability.^69^ Park *et al.* established that the surface wettability as a prime regulator improved osteoblast differentiation, however integrin expression could be governed by both surface microstructure and surface chemistry.^70^ In the present study, the obtained result shows that the cells over all of the PEDOT/ST substrates proliferate energetically and rapidly compared to the cells on PEDOT/UT and uncoated substrates. It is assumed that the PEDOT/ST coatings could offer a dense quantity of cellular binding spots, and additionally, the hydrophilic surface might facilitate tissue cell ingrowth, which are the main targets to improve the cellular action and biocompatibility of implants.

During the valuation of bioimplants, surface features, such as surface topography, surface energy, and wettability properties, play major roles in strongly influencing osteoblast cell adhesion and proliferation.^71^ Particularly, surface wettability could impact the adsorption of cells directly, as the attachment phase as an early stage involves physicochemical links between cells and surfaces, including ionic forces, or indirectly by modifying the attachment of conditioning molecules, *e.g.*, proteins.^72^ Fundamentally, increased wettability improves the interaction between implant surfaces and the physiological environment. In the present study, the contact angles of uncoated and coated TNZ surfaces were found to be 56.70° and 10.40°, respectively (Fig. S8 as ESI†). Hence, PEDOT coatings on treated TNZ substrates might provide an appropriate surface for MG63 cells to efficiently attach and proliferate. Based on the obtained results, we concluded that the PEDOT coatings on treated TNZ substrates could provide suitable surfaces for MG63 cells to attach and proliferate and exhibit better biocompatibility than PEDOT/UT.

## 4 Conclusions

PEDOT coatings have been successfully synthesized on surface-treated TNZ substrates through electrochemical routes. The effects of different surface pretreatments on the surface characteristics, wettability and adhesion of the coatings, corrosion protection in SBF, and *in vitro* biocompatibility were investigated. Surface characterization results revealed the beneficial role of surface treatment before electrodeposition in terms of morphology, topography and hydrophobicity, which facilitates the formation of compact PEDOT films. Interestingly, surface treatment removes the oxide layer and alters the surface morphology, surface roughness, and surface wettability, which increase the surface energy and improve the adhesion strength of the PEDOT coatings prepared on the treated TNZ surface compared to the untreated surface. The results from electrochemical corrosion studies implied that the PEDOT coatings offered improved barrier protection performance when deposited on the surface-treated TNZ substrates. *In vitro* biocompatibility studies on MG63 cells confirmed that cell attachment and proliferation are higher on the surface of the PEDOT coatings prepared on treated TNZ surfaces. Based on the obtained results, it can be concluded that the surface treatment 1 is highly suitable/best ST method to get good quality PEDOT coatings which can be a potential coating material for implant applications.

## Conflicts of interest

There are no conflicts to declare.

## Supplementary Material

RA-008-C8RA01718B-s001
