# Supplementary material for: Influence of surface treatment on PEDOT coatings: surface and electrochemical corrosion aspects of newly developed Ti alloy
Source: RSC Adv. 2018 May 24;8(34):19181–95. doi: 10.1039/c8ra01718b (PMC9080621; doi:10.1039/c8ra01718b)

Electronic Supplementary Material (ESI) for RSC Advances.
This journal is © The Royal Society of Chemistry 2018

Influence of surface treatment on PEDOT coatings: Surface and electrochemical corrosion aspects of newly developed Ti alloy

A.Madhan Kumar[a*], M.A.Hussein[a], Akeem Yusuf Adesina[b], Suresh Ramakrishna[c,d] and N. Al-Aqeeli[b]

[a]Centre of Research Excellence in Corrosion, Research Institute, King Fahd University of Petroleum and Minerals, Dhahran, Saudi Arabia

[b]Department of Mechanical Engineering, King Fahd University of Petroleum and Minerals, Dhahran 31261, Saudi Arabia

[c]Graduate School of Biomedical Science and Engineering, Hanyang University, Seoul, South Korea

[d]College of Medicine, Hanyang University, Seoul, South Korea

Figure S1 Schematic representation of contact angle meausrement in static and dynamic mode.

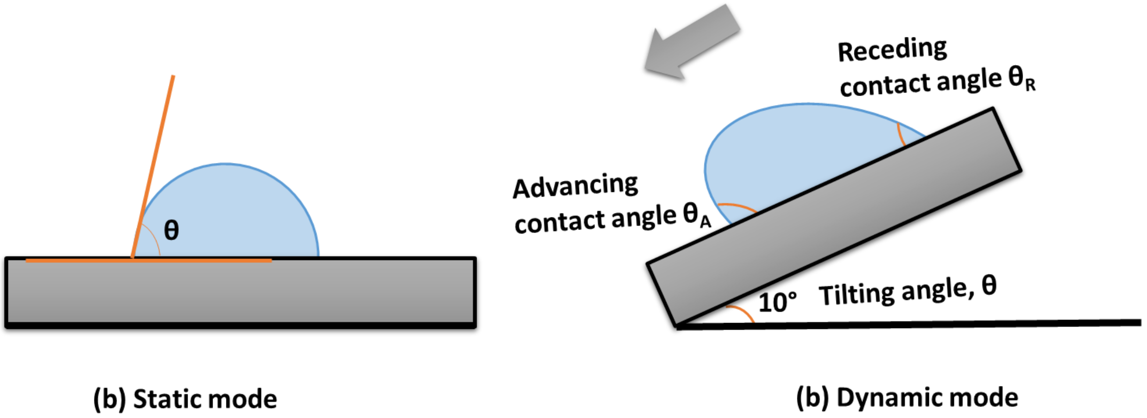

Figure S2 EDS results of (a) Untreated TNZ (b) ST1 (c)ST2 and (d) ST3 TNZ substrates

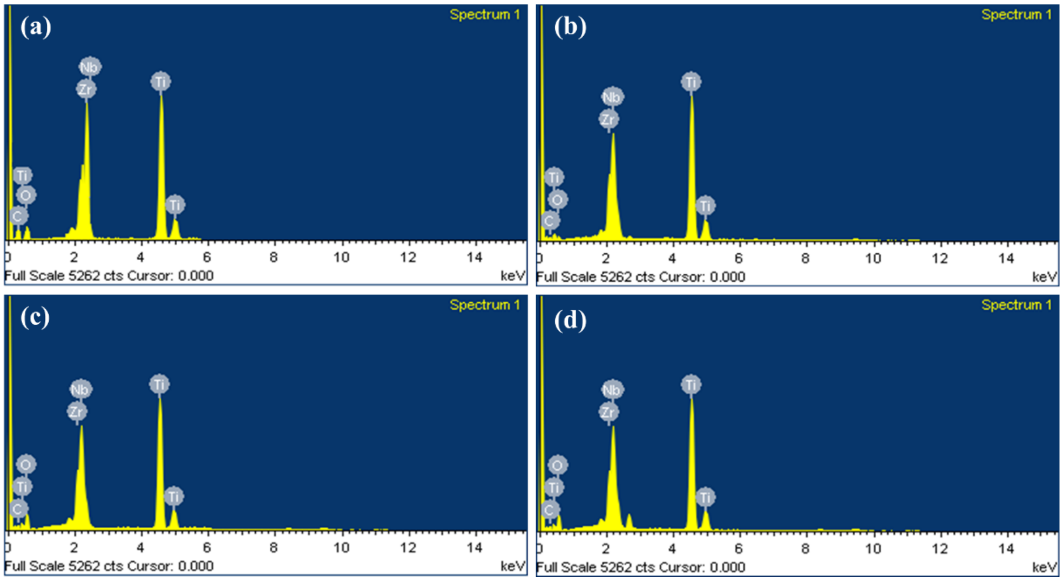

Figure S3 Dynamic contact angle results of (a) Untreated TNZ (b) ST1 (c)ST2 and (d) ST3 TNZ substrates

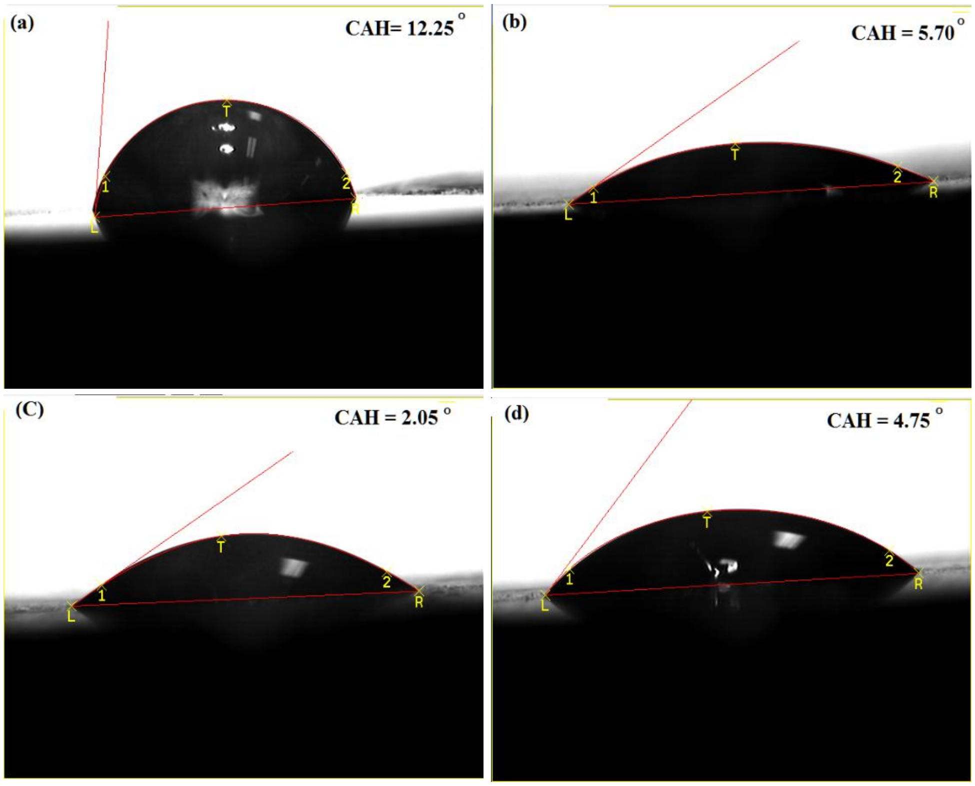

Figure S4 EDS results of (a) PEDOT/UT (b) PEDOT/ST1 (c)PEDOT/ST2 and (d) PEDOT/ST3 TNZ substrates

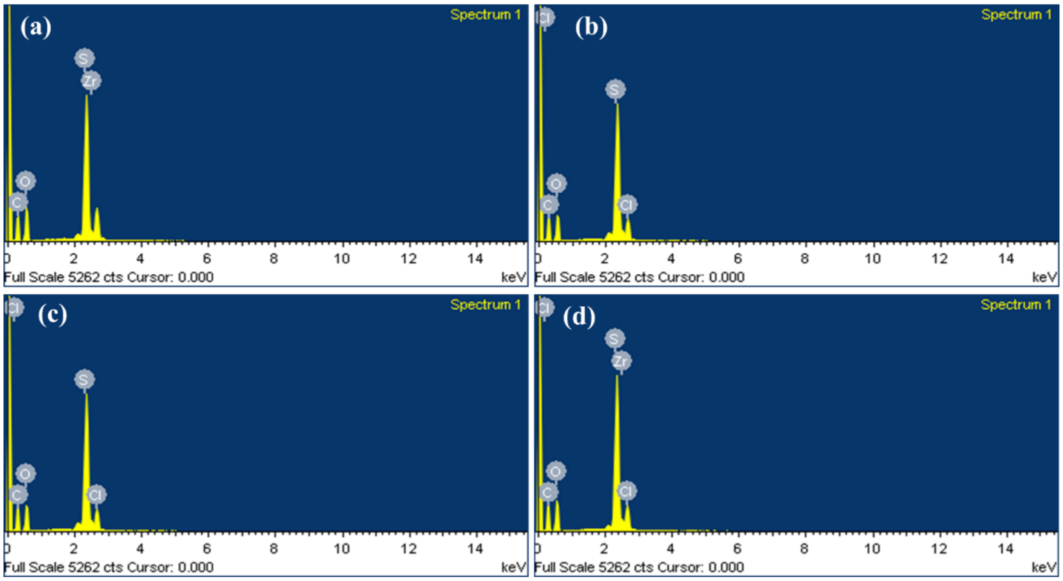

Figure S5 LPR cures of uncoated and coated TNZ substrates

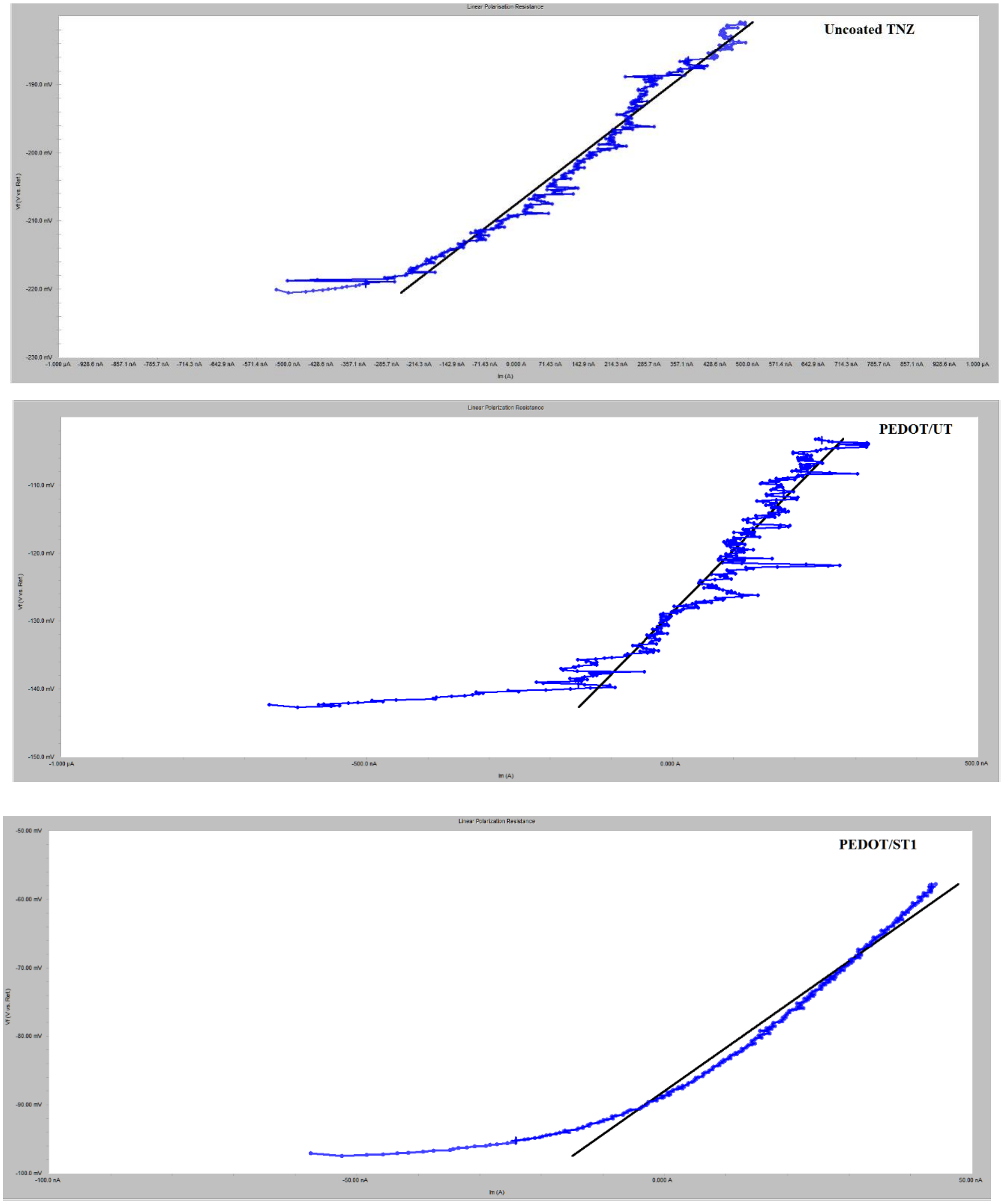

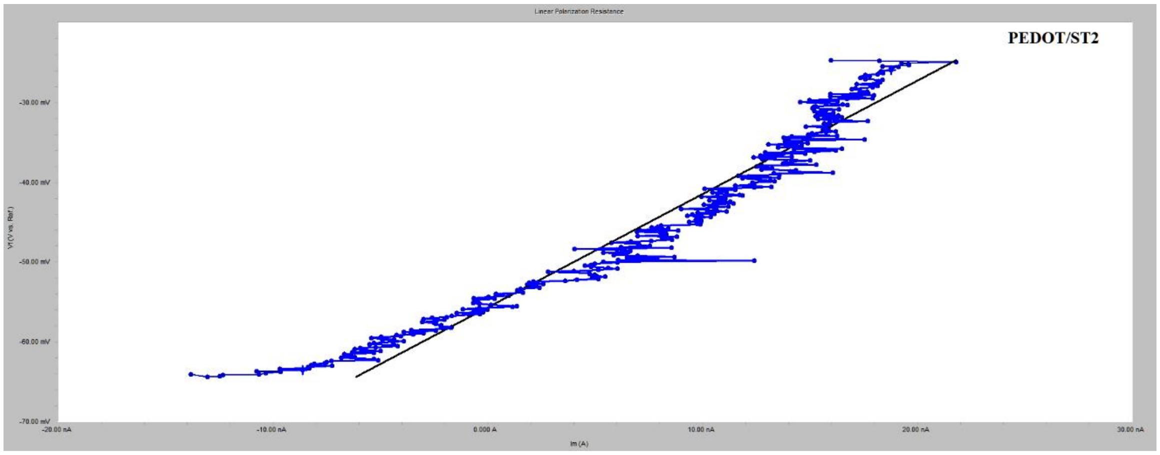
Linear Polarization Resistance
PEDOT/ST2
Vf (V vs. Ref.)
-30.00 mV
-40.00 mV
-50.00 mV
-60.00 mV
-70.00 mV
-20.00 nA
-10.00 nA
0.000 A
10.00 nA
20.00 nA
30.00 nA
Im (A)

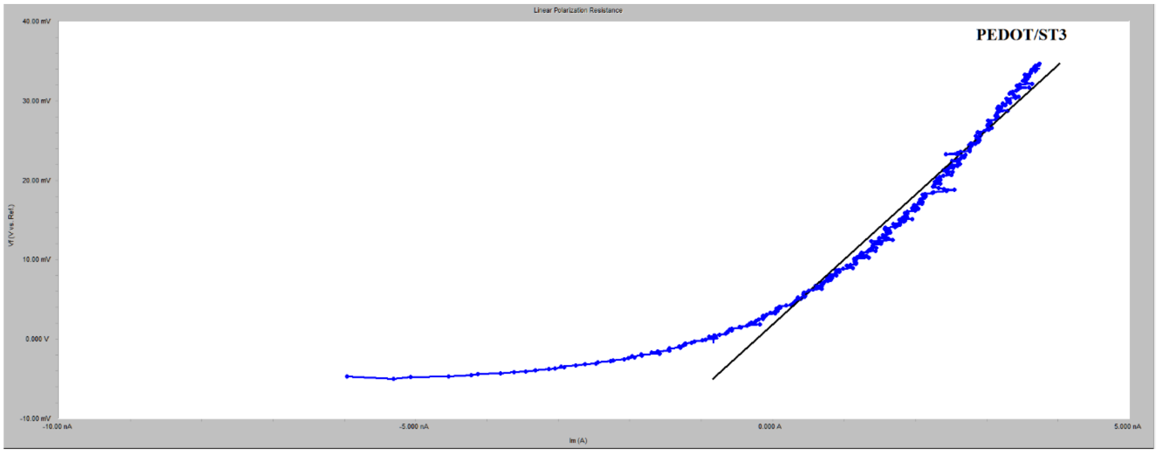
Linear Polarization Resistance
PEDOT/ST3
Vf (V vs. Ref.)
40.00 mV
30.00 mV
20.00 mV
10.00 mV
0.000 V
-10.00 mV
-10.00 nA
-5.000 nA
0.000 A
5.000 nA
Im (A)

Figure S6 EIS circuits of uncoated and coated TNZ substrates

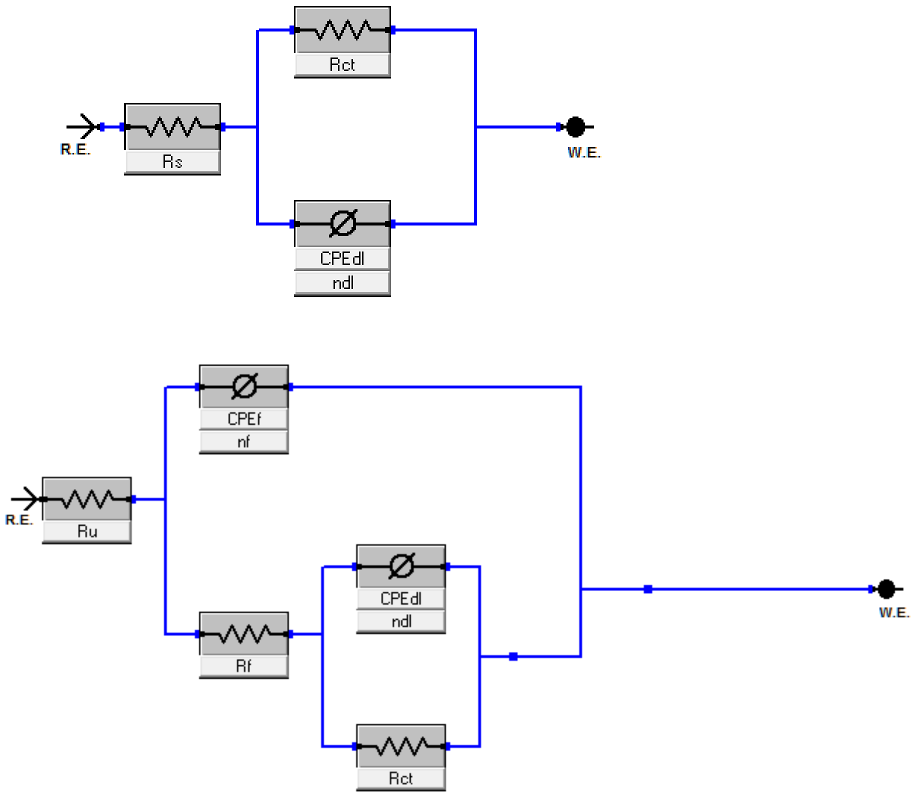

Figure S7 In vitro MG63 osteoblast Cell culture studies of PEDOT coatings on ST2 and ST3 TNZ substrates

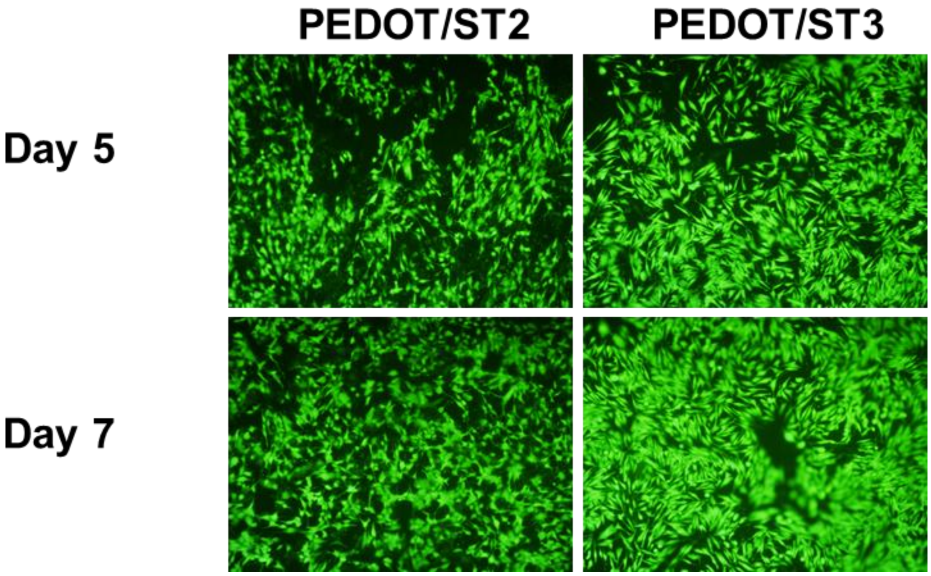

Figure S8 Contact angle images of (a) PEDOT/UT (b) PEDOT/ST1 (c)PEDOT/ST2 and (d) PEDOT/ST3

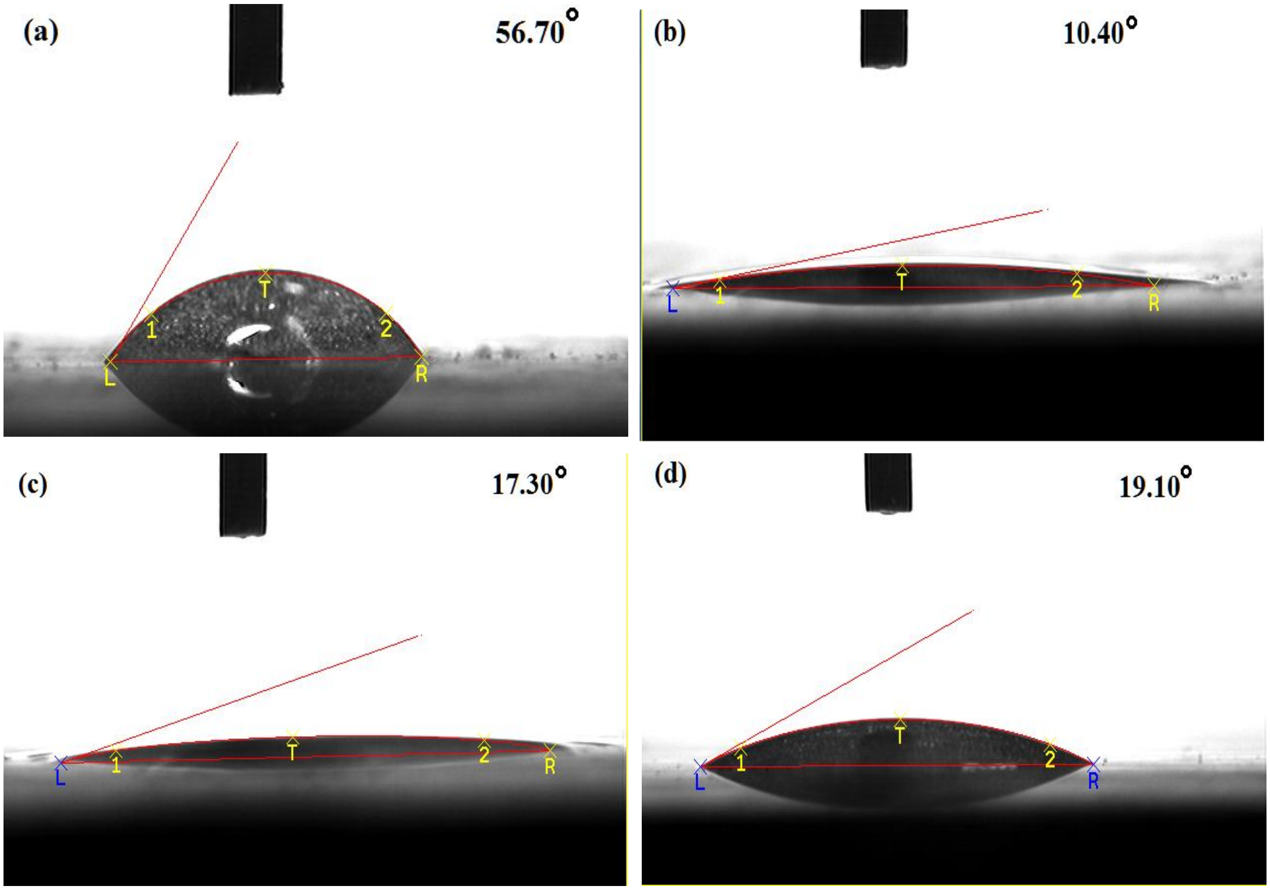

Supplement: RA-008-C8RA01718B-s001 [file RA-008-C8RA01718B-s001.pdf]
